# Integrating QTL mapping and transcriptome analysis to provide molecular insights into gynophore-pod strength in cultivated peanut (*Arachis hypogaea L*.)

**DOI:** 10.3389/fpls.2024.1500281

**Published:** 2024-11-19

**Authors:** Wen Chu, Xiaofeng Zhu, Tao Jiang, Song Wang, Wanli Ni

**Affiliations:** Crops Research Institute, Anhui Academy of Agricultural Sciences, Hefei, Anhui, China

**Keywords:** peanut (*Arachis hypogaea L*.), gynophore-pod strength, QTL, lignin, transcriptome analysis

## Abstract

**Introduction:**

Gynophore-pod strength is one of important mechanical properties that affect mechanized harvesting quality in peanut. Yet its molecular regulation remains elusive.

**Methods:**

We measured gynophore-pod strength across three environments using a recombinant inbred line (RIL) population derived from a cross between Yuanza9102 and Xuzhou68-4, followed by QTL mapping. Lines with extreme gynophore-pod strength from the RILs were selected to perform anatomical analysis and transcriptome analysis to elucidate the underlying molecular mechanisms governing gynophore-pod strength.

**Results and discussion:**

Both genotypic factor and environments affected gynophore-pod strength significantly, and its broad sense heritability (*h^2^*) was estimated as 0.77. Two QTLs that were stable in at least two environments were detected. *qGPS.A05-1* was mapped 4cM (about 1.09Mb) on chromosome A05, and *qGPS.B02-1* was mapped 3cM (about 1.71Mb) on chromosome B02. Anatomical analysis showed higher lignin content in lines with extreme high gynophore-pod strength compared to those with extreme low gynophore-pod strength. Additionally, comparative transcriptome analysis unveiled that phenylpropanoid biosynthesis was the main pathway associated with high gynophore-pod strength. Further, we predicted *VJ8B3Q* and *H82QG0* as the candidate genes for *qGPS.A05-1* and *qGPS.B02-1*, respectively. The two stable QTLs and their associated markers could help modify gynophore-pod strength. Our findings may offer genetic resources for the molecular-assisted breeding of new peanut varieties with improved mechanized harvesting quality.

## 1 Introduction

Cultivated peanut (*Arachis hypogaea L*.) is an allotetraploid (AABB, 2n = 4x =40), which is assumed originated from a hybridization event between *A. duranensis* (AA, 2n = 2x = 20) and *A. ipaensis* (BB, 2n = 2x = 20) (Moretzsohn et al., 2013; Bertioli et al., 2019). As one of the most promising oil crops, peanuts are cultivated in over 100 countries. Globally, peanut is cultivated in over 32.7 million hectares, and approximately 53.9 million tons of annual production (Food and Agriculture Organization of the United Nations, 2024). In addition to being a source of high-quality cooking oil and edible nuts, peanuts also serve as an excellent livestock feed due to rich nutrients in their aerial parts.

Peanut produce aerial flowers but subterranean fruits (pods). Embryo development stalled until the fertilized ovary is buried in the soil with the help of a specialized organ called the peg or gynophore. Since mature pods are underground, they must be dug out during harvest. Consequently, the mechanical properties of the gynophore-pod, gynophore, and branch-gynophore are crucial for the quality of mechanized harvesting. Among them, strength of gynophore-pod is the lowest, primarily leading to yield loss during mechanized harvesting. Thus, understanding the mechanism behind gynophore-pod strength is essential for enhancing the quality of mechanized peanut harvesting.

Lignin is a principal structural component of cell walls in plants, which reinforce the cell wall and provide mechanical strength for tissues (Ralph et al., 2019; Vanholme et al., 2019). Higher lignin content of stems correlates positively with stem strength (Berry et al., 2004; Kong et al., 2013; Peng et al., 2014). Abnormal lignin deposition in the abscission layer (Wu et al., 2017; Lv et al., 2018) or dehiscence zone (Dong et al., 2014) contributes to shatter resistance. Genetic modification of lignin in specific tissues has been one of crucial strategies for altering crop traits like lodging and shattering. Lignin is derived primarily from oxidative coupling of three phenylpropanoid monomeric precursors: *p*-coumaryl, coniferyl and sinapyl alcohols, which form into *p*-hydroxyphenyl (H), guaiacyl (G) and syringyl (S) units, respectively (Liu et al., 2018; Ralph et al., 2019). Several key genes involved in the biosynthesis of monolignols in the phenylpropanoid pathway include phenylalanine ammonia lyase (*PAL*), cinnamate 4-hydroxylase (*C4H*), 4-coumarate: CoA ligase (*4CL*), *p*-coumarate 3-hyroxylase (*C3H*), ferulate-5-hydroxylase (*F5H*), caffeic acid 3-O-methyltransferase (*COMT*), caffeoyl-CoA O-methyltransferase (*CCoAOMT*) and cinnamoyl-CoA reductase (*CCR*) (Vanholme et al., 2019; Zhong et al., 2019).

With the help of many peanut genetic linkage maps, more and more quantitative trait locus (QTL) associated with complex quantitative traits were identified. For instance, a major QTL, *cqSPA09*, for shelling percentage was identified in a recombinant inbred line (RIL) population from the cross of Yuanza9102 and Xuzhou68-4 (Luo et al., 2017). three main-effect QTL for initial flowering date were identified by using a RIL population derived from the cross between Silihong and Jinonghei3 (Wang et al., 2020); a stable and major QTL for pod shape was identified in a RIL population development from a cross between Huayu36 and 6-13 (Zhang et al., 2023). RNA sequencing has become a powerful high-throughput method for analyzing gene expression across the entire transcriptome. It has been successfully applied to explore genes involved in secondary metabolite biosynthesis in plants (Wong et al., 2011; Zhu et al., 2023).

In this study, we used a RIL population derived from the cross between Yuanza9102 and Xuzhou68-4 (Luo et al., 2017) to detected QTLs for gynophore-pod strength. We analyzed the anatomical features of gynophore-pod nodes from extreme trait lines to reveal the cellular mechanisms underlying gynophore-pod strength. In addition, we performed comparative transcriptome sequencing of extreme trait lines to reveal key metabolic pathways associate the gynophore-pod strength. Our results may trigger functional genomic studies toward the molecular-assisted breeding of new peanut varieties with higher mechanized harvesting quality.

## 2 Materials and methods

### 2.1 Plant materials and growth conditions

A mapping population comprising 195 F_8_ recombinant inbred lines (RILs), derived from the Yuanza9102 × Xuzhou68-4, was obtained from the Oil Crops Research Institute, Chinese Academy of Agricultural Sciences (OCRI-CAAS) (Luo et al., 2017). The F_9_-F_11_ RILs and their parents were planted in the experimental field in Guzhen (GZ; 33°14′N, 117°19′E), Bengbu, China, in three consecutive years from 2022 to 2024. These experiments were treated as three environments and designated as Guzhen2022, Guzhen2023 and Guzhen2024 in this study. In each environment, the parents and the RIL population were planted in a randomized complete block design with two replications. Each plot contained two rows, with 10 plants in each row, 10 cm between plants and 25 cm between rows. Standard field management practices were followed.

### 2.2 Phenotypic evaluations and data analysis

After harvesting, gynophore will dehydrate and become brittle within a few days, making it impossible to assess gynophore-pod strength using dehydrated samples. In addition, the gynophore-pod strength varies significantly with changes in water content of gynophore-pod samples. So proper storage conditions for gynophore-pod samples are crucial for obtaining accurate measurements. For the storage test, well developed gynophore-pods (including pod and gynophore) from Yuanza9102 and Xuzhou 68-4 were collected 10 days before harvesting from five representative plants and stored in sealed plastic bags, with 50 gynophore-pods per bag, at 25 °C, 4 °C, and -20°C, respectively. A control group of gynophore-pods from Yuanza9102 and Xuzhou 68-4 was also stored unsealed at 25 °C. The strength of the gynophore-pods was measured at 0, 3, 6, 9, and 12 days using an HP-500N digital force gauge (Yueqing Handpi Instruments Co, Zhejiang, China). Two terminals of each gynophore-pod were clipped to the force gauge, and the maximum load required to break the gynophore-pod node was recorded. For phenotypic evaluations of the RIL population, all gynophore-pods were stored in sealed plastic bags at 4 °C. To ensure consistency, all gynophore-pods strength test were completed within 6 days of harvesting. Average value of five gynophore-pod strength of each line was considered as its gynophore-pod strength.

The one-way Analysis of variance (ANOVA) and Duncan’s *post hoc* test for the phenotypic data of gynophore-pod strength were conducted using SPASS 20.0 software (SPSS, Inc., IL, USA). Statistically significant differences were set at *p* value ≤ 0.05. Broad-sense heritability (*h* ^2^) and best linear unbiased estimation (BLUE) values were performed using the ANOVA of multi-environmental trials in QTL IciMapping 4.2 (Wang, 2009; Meng et al., 2015). The broad-sense heritability was estimated using the formula: $h^{2}=\;\sigma_{ɡ}^{2}/\left(\sigma_{ɡ}^{2}\;+\;\sigma_{ɡ\times e}^{2}/r+\;\sigma_{e}^{2}/rn\right)$ , where $\sigma_{ɡ}^{2}$ is the genotypic variance, $\sigma_{ɡ\times e}^{2}$ is variance of interaction between genotype and environment, $\sigma_{e}^{2}$ is the error variance, and r is the number of environment trails, n is number of replications in each field trails.

### 2.3 QTL mapping

The parents and RILs were previously genotyped (Luo et al., 2017). The constructed genetic linkage map measures 1386.19 cM and harbors 830 SSR markers distributed on 20 linkage groups with an average inter-markers distance of 1.67 cM (Luo et al., 2017). We conducted the QTL mapping using the inclusive composite interval mapping (ICIM) method in IciMapping V4.2 software (Wang, 2009; Meng et al., 2015). For the phenotypic data in single environment and the BLUE value, QTLs were analyzed using the ICIM-ADD mapping method. The specific parameters were set as: Step = 1.0 cM, value p for input variables (PIN) = 0.001, and logarithm of odds (LOD) =2.5. Each LOD score larger than 2.5 was considered as resulting from the presence of a QTL. QTLs are named “*q*” + “abbreviation of the specific trait” + “specific linkage group” + “-” + “number”. QTLs which detected in different environments had overlapping 2-LOD support intervals were considered to be a consistent QTL.

### 2.4 Anatomical analysis

Two lines (line137 and line 151) from extreme high gynophore-pod strength group and two lines (line 172 and line 173) from extreme low gynophore-pod strength group were selected to perform anatomical analysis. Their gynophore-pod nodes of 10 days before harvest were collected and fixed in FAA solution (50% ethanol, acetic acid, 3.7% formaldehyde) for 1 day. Fixed samples were dehydrated in an ethanol series and embedded in paraffin wax. Longitudinal sections were further sliced and stained with 0.1% toluidine blue. For visualization of lignified tissues, the paraffin sections were placed in 50 mL sterile tubes containing 2% (v/v) phloroglucinol for 5 min, then paraffin sections were added drops of concentrated HCL to cause the Wiesner reaction. Photographs were taken with an Olympus CX51 microscope (Olympus, Tokyo, Japan) with Sony digital camera (Sony, Tokyo, Japan).

### 2.5 RNA−seq analysis

Lines 137 and 173, representing the extreme trait group with contrasting genotypes at the consistent QTLs *qGPS.B02-1* and *qGPS.B02-1*, were selected and subjected to transcriptome analysis at 20 days before harvest. Anatomical analysis showed lignin content was significant differences in gynophore-pod node between the extreme lines. Therefore, we performed transcriptome analysis using gynophore-pod node which included 1mm pod end and 5mm gynophore. Total RNA was extracted using RNA Easy Fast kit (Tiangen Biotech, Beijing, China) to construct independent RNA libraries with three replicates for each line. The RNA libraries were then subjected to RNA sequencing at Biomarker Technologies Co. (Beijing, China). The low-quality reads including adapter and reads containing ploy-N were excluded using fastp preprocessor (Chen et al., 2018). After removal of low-quality reads, clean reads were aligned to the Tifrunner reference genome (https://data.legumeinfo.org/Arachis/hypogaea/genomes/Tifrunner.gnm2.J5K5/arahy.Tifrunner.gnm2.J5K5.genome_main_softmasked.fna.gz) with TopHat2 (Kim et al., 2015). The mapped reads of each sample were assembled by StringTie in a reference-based approach (Pertea et al., 2015). FPKM (Fragments Per Kilobase of transcript per Million fragments mapped) of each gene was calculated based on the length of the gene and reads count mapped to this gene using StringTie (Pertea et al., 2015). DEGs (differentially expressed genes) analysis was performed using DESeq2 R package (Love et al., 2014). Significant DEGs were identified at a false discovery rate (FDR) ≤ 0.01 and |log2(FoldChange)| ≥ 1. GO (Gene ontology) and KEGG (Kyoto Encyclopedia of Genes and Genomes) enrichment analyses of DEGs were conducted in clusterProfiler (Young et al., 2010).

### 2.6 Real−time PCR

The RNA which subjected to performed transcriptome analysis also used to perform RT-PCR. The synthesis of first-strand cDNA was performed using HiScript III 1st Strand cDNA Synthesis Kit (Vazyme, Nanjing, China). The RT-PCR was achieved with three biological and technical repeats in the CFX Connect Real-Time PCR Detection System (Bio-Rad, California, USA) using Super-Real PreMix Plus (SYBR Green; Vazyme, Nanjing, China). All primers were designed using Primer 5 software and synthesized by Tsingke Biotech Co. (Beijng, China). The *Actin* gene from peanut (*A. hypogaea*) (Zhu et al., 2021) was used as an internal control and all primers are listed in **Supplementary Table S1**. The quantified data were calculated using the 2^−ΔΔCt^ method.

## 3 Results

### 3.1 Gynophore-pod strength phenotyping of RIL population

As shown in **Table 1**, gynophore-pod strength of Yuanza9102 remained consistent across all storage condition for 3 days. After 6 days, significant variations were noted at all conditions except 4 °C. For Xuzhou68-4, gynophore-pod strength did not significantly change at 4 °C and -20 °C for 3days, nor at 4 °C for 6 days. Ultimately, to obtain accurate measurement, all RILs samples were stored in sealed bags at 4 °C, and gynophore-pod strength tests were conducted within 6 days of harvesting.

**Table 1:** Impact of gynophore-pod strength under various storage conditions.

Storage conditions	Line	gynophore-pod strength (N)			
		0d	3d	6d	9d
CK	Yuanza9102	10.94a	10.54a	4.74b	5.28b
	Xuzhou68-4	12.84a	8.76b	8.58b	8.4b
25°C	Yuanza9102	10.94a	11.02a	9.48b	–
	Xuzhou68-4	12.84a	10.50b	7.84b	–
4°C	Yuanza9102	10.94a	11.68a	11.38a	11.77a
	Xuzhou68-4	12.84a	11.94a	12.00a	10.78b
-20°C	Yuanza9102	10.94a	11.32a	10.46b	9.95b
	Xuzhou68-4	12.84a	11.96a	8.14b	6.84c

CK gynophore-pods were stored unsealed at 25 °C, *‘–’* indicates no data. Lowercase letters in the column show significant difference (p < 0.05).

**Figure 1:**
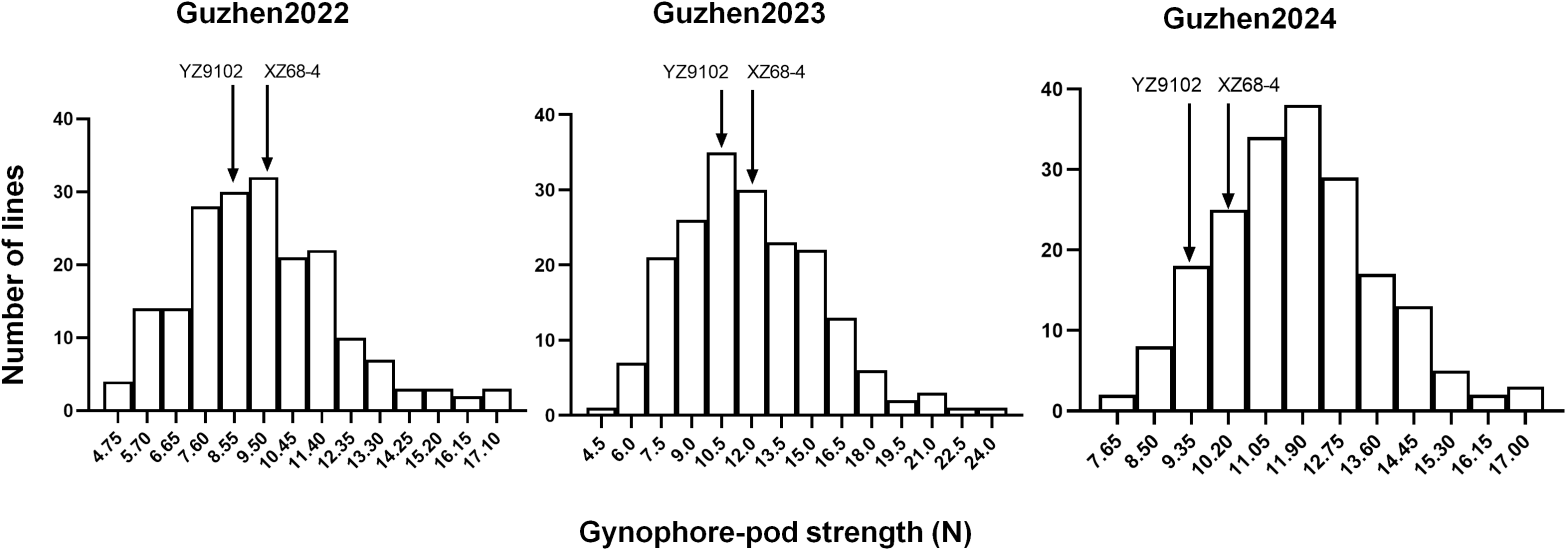
Phenotypic distribution of gynophore-pod strength in the RIL population across 3 years. The YZ9102 represented the gynophore-pod strength of Yuanza9102, and the XZ68-4 represented the gynophore-pod strength of Xuzhou68-4.

The summary of the descriptive statistic of gynophore-pod strength of RILs in the three different environments is presented in **Table 2**. In general, gynophore-pod strength ranged from 9.47 ± 2.53 N to 11.85 ± 3.50 N, with a CV (coefficient of variation) of 16%-30% (**Table 2**). The gynophore-pod strength in the RIL population showed a continuous distribution in each environment, indicating polygenic inheritance (**Table 2**; **Figure 1**). The value of broad-sense heritability for gynophore-pod strength was estimated to be 0.77 (**Table 2**), indicating that the phenotypic variance was strongly controlled by genetic factors. Variance analysis across the three trials also revealed that genetic, environmental effects and genotype × environment interactions significantly influenced gynophore-pod strength, and gynophore-pod strength was influenced more by the environment than by genotype × environment (**Table 3**).

**Table 2:** Phenotypic variation characteristics of gynophore-pod strength of RIL population in different environments.

Year	Parents		RILs							
	P1	P2	Min	Max	Mean	SD	CV (%)	Kurt	Skew	*h^2^*
2022	8.40	9.76	4.66	17.39	9.47	2.53	27	0.57	0.67	0.77
2023	11.60	12.91	5.04	24.08	11.85	3.50	30	0.48	0.65	
2024	9.40	10.58	7.26	17.21	11.76	1.85	16	0.16	0.35	

P1 female parent Yuanza9102, P2 male parent Xuzhou68-4, Min minimum, Max maximum, Mean mean trait value, SD standard deviation, CV coefficient of variation, Kurt kurtosis, Skew skewness, h^2^ heritability.

**Table 3:** Variance analysis for gynophore-pod strength in the RIL population in three environments.

Variables	*df*	Mean square	*F* value	*P* value
Environment	2	771.31	291.86	< 0.001
Genotype	194	28.21	10.67	<0.001
Genotype × environment	382	7.67	2.9	<0.001
Error	576	2.64		

### 3.2 Detection of QTLs for gynophore-pod strength

The phenotypic data of gynophore-pod strength from the three different environments and previously constructed genetic linkage map (Luo et al., 2017) were used for QTL mapping. A total of 10 QTLs for gynophore-pod strength were detected (**Table 4**; **Supplementary Figure S1**). These QTLs were located on chromosome A05, A08, B02, B04 and B05, with a PVE (phenotypic variation explained) ranging from 3.90 to 9.57%. *qGPS.A05-1*, located at a 4 cM interval on chromosome A05, was detected in all environments, with a PVE ranging from 3.90 to 6.44%. The additive genetic effect of *qGPS.A05-1* was positive, indicating the allele enhancing gynophore-pod strength came from the parent Xuzhou68-4. *qGPS.B02-1*, located at a 3 cM interval on chromosome B02, was also detected in two environments (2022Guzhen and 2024Guzhen), with a PVE of 5.71% and 8.20%, respectively. However, the additive genetic effects were negative, suggesting that Yuanza9102 contributed the allele improving gynophore-pod strength. *qGPS.A08-1*, *qGPS.B02-2*, *qGPS.B04-1*, *qGPS.B04-2* and *qGPS.B05-1* were only detected in one environment, and all of them had negative additive genetic effects, which also indicates that the alleles for increasing gynophore-pod strength came from the parent Yuanza9102. In addition, using the BLUE value of gynophore-pod strength of every RIL across three environments, two QTLs (*qGPS.A05-1* and *qGPS.B02-1*) have been detected. And they have been detected with the phenotypic data of gynophore-pod strength under single environment.

**Table 4:** QTL mapping for gynophore-pod strength in RIL population.

QTL Locus	Environment	LG	CI (cM)	POS (cM)	LOD	Additive	PVE (%)
*qGPS.A05-1*	2022Guzhen	A05	99.5-102.5	102	4.25	0.70	6.44
	2023Guzhen	A05	99.5-102.5	102	2.72	0.85	3.90
	2024Guzhen	A05	98.5-102.5	102	3.14	0.51	5.71
	BLUE	A05	99.5-102.5	102	4.07	0.59	7.12
*qGPS.A08-1*	2022Guzhen	A08	24.5-45.5	35	3.04	-0.74	7.27
*qGPS.B02-1*	2022Guzhen	B02	84.5-86.5	86	3.77	-0.66	5.71
	2024Guzhen	B02	85.5-87.5	87	4.37	-0.60	8.20
	BLUE	B02	85.5-87.5	87	5.55	-0.69	10.02
*qGPS.B02-2*	2023Guzhen	B02	81.5-83.5	83	5.4	-1.32	9.57
*qGPS.B04-1*	2022Guzhen	B04	62.5-64.5	64	2.94	-0.62	4.91
*qGPS.B04-2*	2023Guzhen	B04	39.5-41.5	41	4.16	-1.24	7.87
*qGPS.B05-1*	2022Guzhen	B05	87.5-89.5	89	2.69	-0.61	4.66

LG linkage group, POS position, CI 2-LODconfidence interval, LOD logarithm of odds, PVE phenotypic variation explained, BLUE best linear unbiased estimation.

### 3.3 Lignin content was significant different between lines with extreme gynophore-pod strength

**Figure 2:**
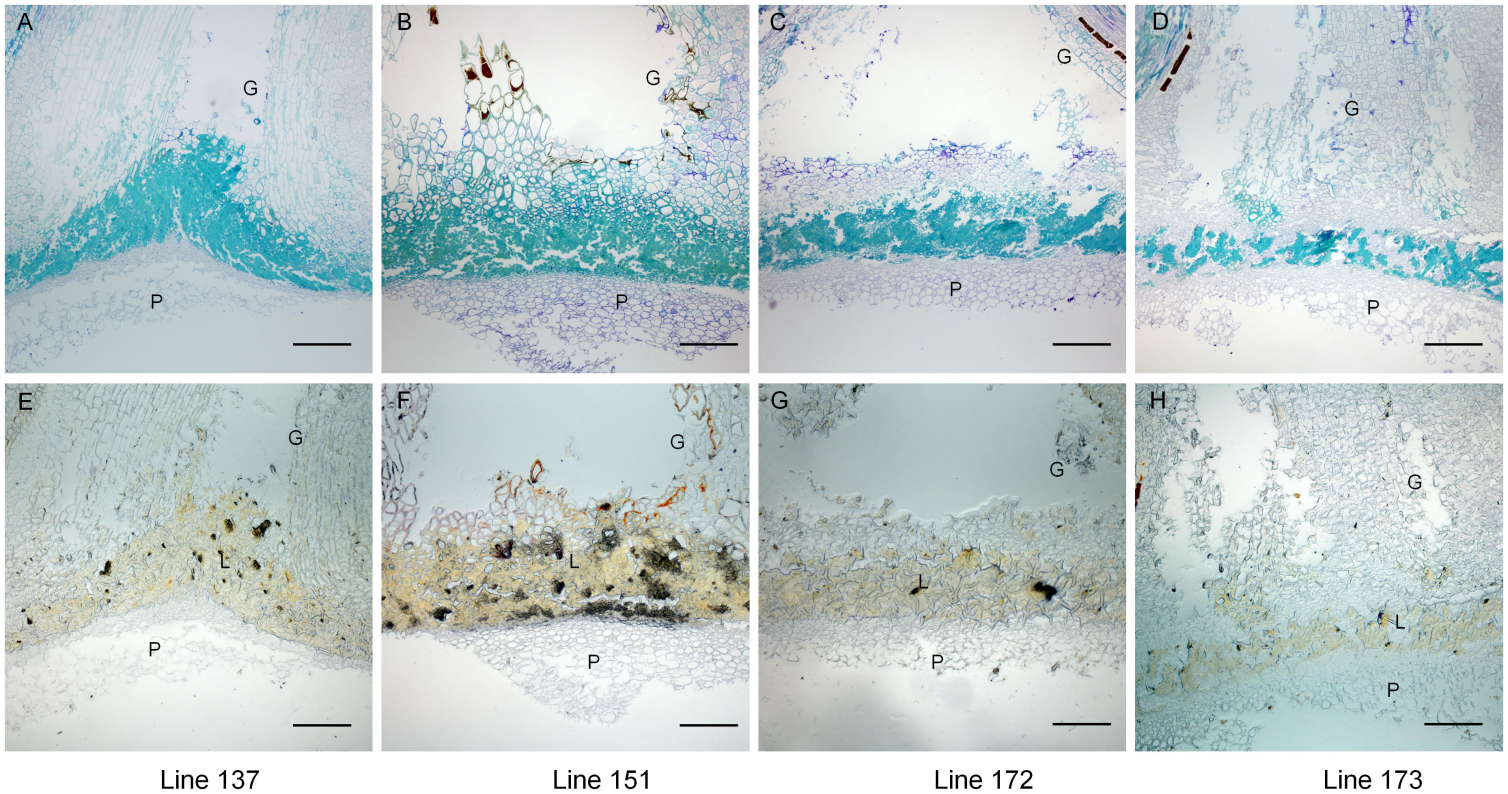
Lines with high gynophore-pod strength exhibited higher lignin content than those with low gynophore-pod strength. Longitudinal sections of toluidine blue-stained gynophore-pod nodes **(A–D)** and those treated with phloroglucinol-HCL **(E–H)**. Line 137 and line 151 exhibited high gynophore-pod strength, while line 172 and line73 exhibited low gynophore-pod strength. G: gynophore; P: pod; L: lignin. Scale bars = 200 μm.

To analyze the cellular mechanisms of gynophore-pod strength between extreme trait lines, we examined the anatomical features of gynophore-pod nodes from two high gynophore-pod strength lines (line137 and line 151) and two low gynophore-pod strength lines (line 172 and line 173) (**Supplementary Figure S2**). As shown in **Figures 2A–D**, sclerenchyma cells were present at the gynophore-pod junction, with significantly more sclerenchyma cells in line 137 and 151 compared to line172 and173. To visualize lignified cells phloroglucinol-HCL treatment were performed. As shown in **Figures 2E–H**, the sclerenchyma cells were lignin-stained. The area and intensity of lignin signal in lines172 and 173 were largely reduced compared to that in lines137 and 151. These results showed that lignin content in gynophore-pod nodes was significantly higher in lines137 and 151 compared to lines 172 and 173.

### 3.4 Transcriptome analysis of lines with extreme gynophore-pod strength

To unveil the molecular mechanisms involved in gynophore-pod strength, transcriptome analysis was performed using gynophore-pod nodes from line137 with high gynophore-pod strength and line 173 with low gynophore-pod strength (**Supplementary Figure S2**). The summary of the sequencing data was presented in **Supplementary Table S2**. The Q20, Q30, and GC content indicate high-quality sequencing data, with over 94.10% of clean reads mapped to the Tifrunner genome (**Supplementary Table S2**). A set of 3512 genes showed differential expression (**Supplementary Figure S3**). To verify the reliability of transcriptome data of lines with extreme gynophore-pod strength values, we randomly selected eight DEGs for RT-PCR analysis. The results showed that the RT-PCR expression data were consistent with the RNA-seq data (**Supplementary Figure S4**), confirming the reliability of the transcriptome data.

**Figure 3:**
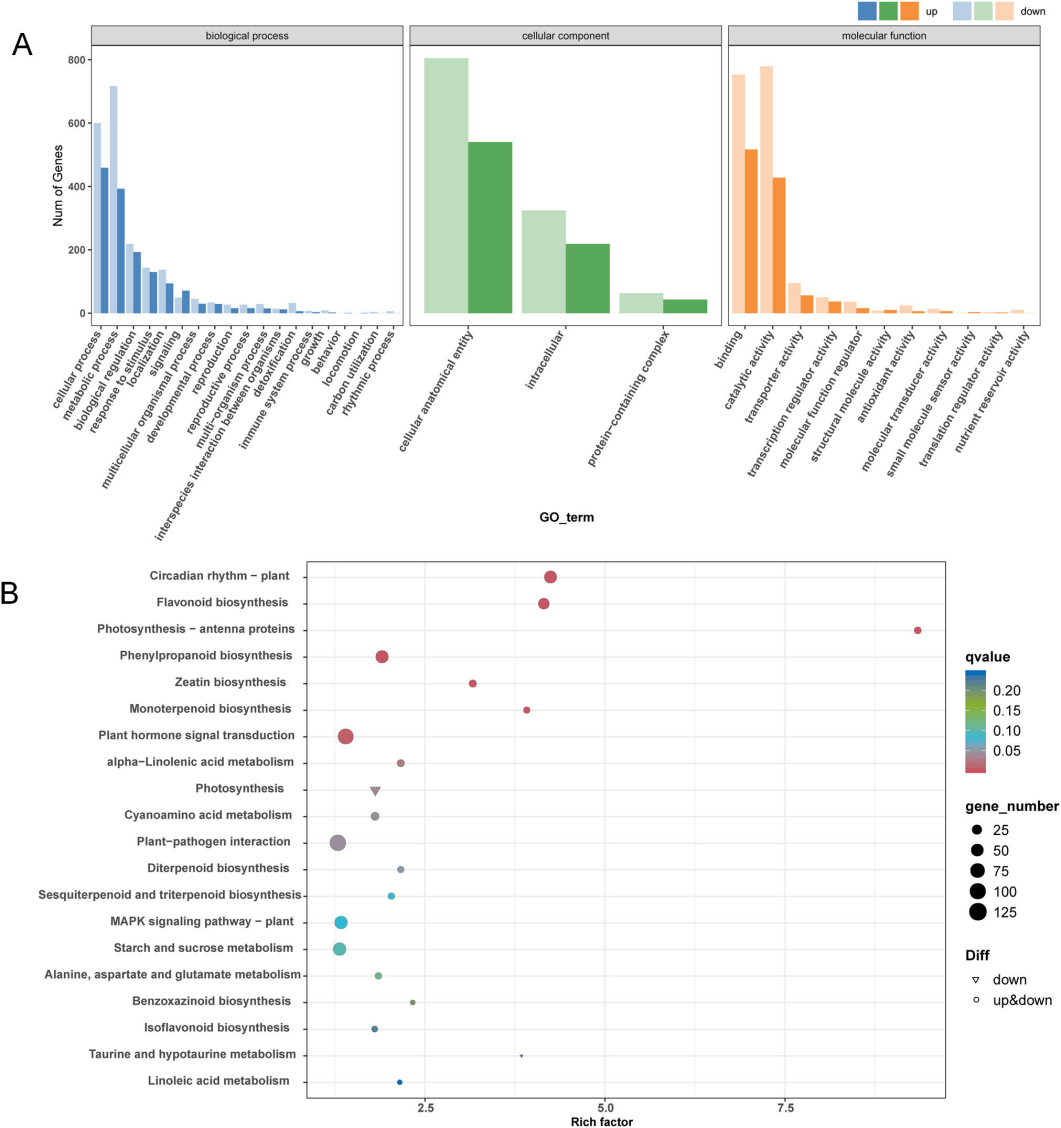
GO function and KEGG pathway analysis of DEGs in line 137 and line 173. GO classification **(A)** and KEGG enrichment **(B)** of DEGs in line 137 and line 173.

**Figure 4:**
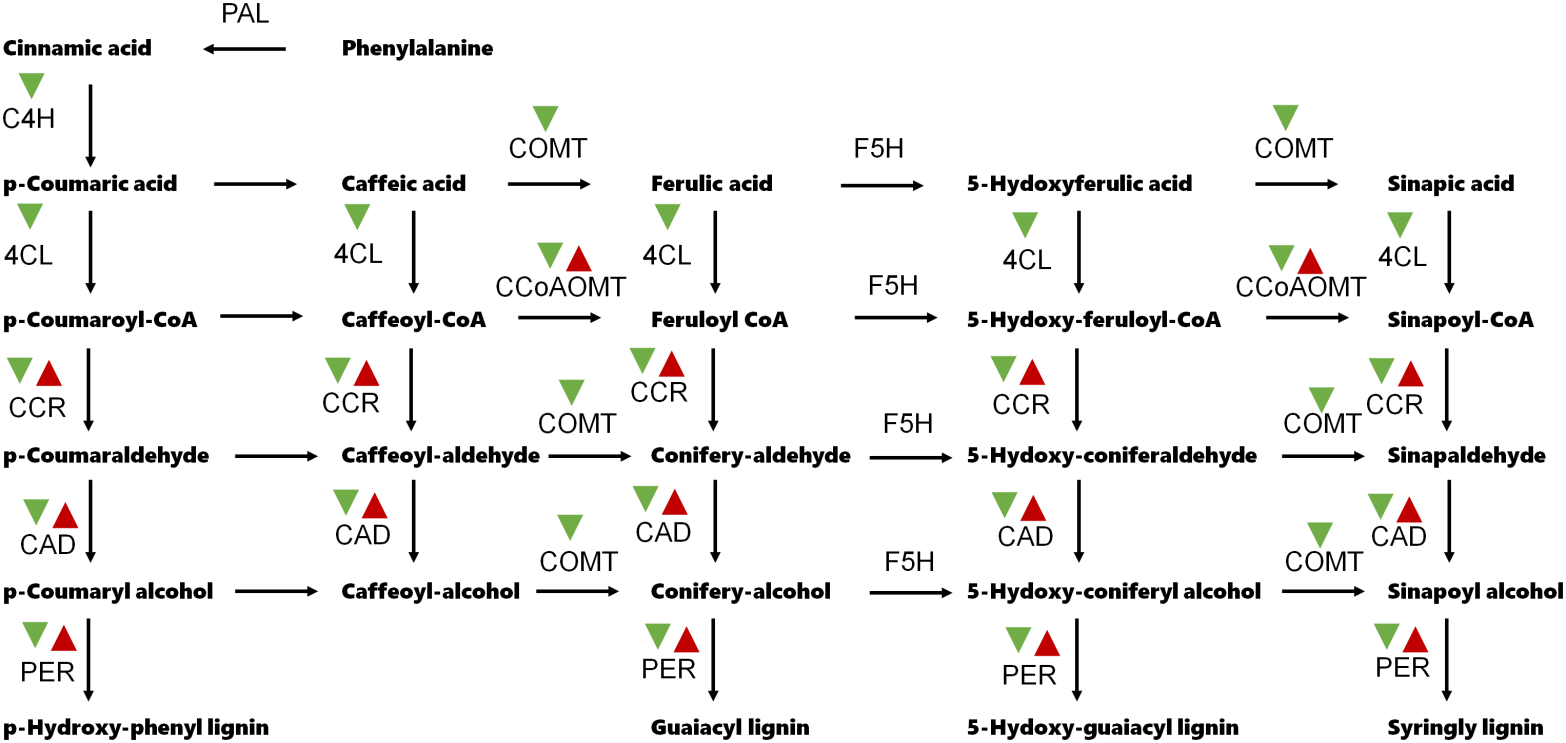
Expression patterns of the phenylpropanoid biosynthesis-related genes. PAL, Phenylalanine ammonia-lyase; C4H, Cinnamate 4-Hydroxylase; 4CL, 4-coumarate-CoA ligase; CCR, Cinnamoyl-CoA reductase; CCoAOMT, caffeoyl-coenzyme A O-methyltransferase; COMT, Caffeic acid 3-O-methyltransferase; CAD, Cinnamyl alcohol deaminase; F5H, Ferulate 5-hydroxylase; PER, Peroxidase. Red and green arrow head indicate up-regulation and down-regulation, respectively.

GO and KEGG analyses were proceeded to understand the main functional classification of the DEGs. According to the result of GO analysis, GO terms related to cell wall development were significantly enriched. Such as cell wall organization, cell wall biogenesis, cellulose biosynthetic process and plant-type secondary cell wall biogenesis in the biological process category; cell wall and plant type cell wall in the cellular component; monooxygenase activity, UDP-glycosyltransferase activity and O-methyltransferase activity in the molecular function (**Figure 3A** and **Supplementary Table S3**). KEGG analysis indicated that flavonoid biosynthesis and phenylpropanoid biosynthesis were significantly enriched. The phenylpropanoid biosynthesis pathway is the main route of lignin synthesis (**Figure 3B** and **Supplementary Table S4**). Several phenylpropanoid biosynthesis-related genes, such as *C4H* (Cinnamate 4-Hydroxylase), *4CL* (4-coumarate-CoA ligase) and *COMT* (Caffeic acid 3-O-methyltransferase), were down-regulated in line 173 (**Figure 4**).

### 3.5 Candidate genes prediction

To explore candidate genes for *qGPS.A05-1* and *qGPS.B02-1*, their border marker primer sequences were aligned to Tifrunner reference sequences to determine their physical intervals (**Table 5**). *qGPS.A05-1* was mapped between the markers AHGS1507 and AhTE0839, which corresponds to 7.35-8.44 Mb in the reference genome. *qGPS.B02-1* was located between the markers TC9F04 and Ai02B2350 corresponding to 4.51-6.22 Mb. There were 6 genes exhibited different expression in the map interval of *qGPS.A05-1* (**Table 6**). Among them, *VJ8B3Q* encodes a DREB protein, from the DREB subfamily A-5 of the ERF/AP2 transcription factor family, and is hypothesized to be linked to phenylpropanoid biosynthesis (Liang et al., 2024). In the map interval of *qGPS.B02-1*, 7 DEGs were identified (**Table 6**). *H82QG0* which encodes a member of CYP98A cytochrome P450 monooxygenase protein family was hypothesized to be associated with phenylpropanoid biosynthesis (Gou et al., 2018). So, *VJ8B3Q* and *H82QG0* were predicted as the candidate genes for *qGPS.A05-1* and *qGPS.B02-1*, respectively.

**Table 5:** Physical interval for *qGPS.A05-1* and *qGPS.B02-1*.

QTL Locus	Environment	Chr	Left Marker	Right Marker	PI (Mb)
*qGPS.A05-1*	2022Guzhen	A05	AHGS1507	AhTE0839	7.35-8.44
	2023Guzhen	A05	AHGS1507	AhTE0839	7.35-8.44
	2024Guzhen	A05	AHGS1507	AhTE0839	7.35-8.44
*qGPS.B02-1*	2022Guzhen	B02	Ai02B2350	AHGS1940	5.52-6.22
	2024Guzhen	B02	AHGS1940	TC9F04	4.51-5.52

Chr chromosome, PI physical interval.

**Table 6:** Information of genes differentially expressed between line137 and line173 within the mapping intervals of *qGPS.A05-1* and *qGPS.B02-1*.

QTL	ID	log_2_FC	Description
*qGPS.A05-1*	*VJ8B3Q*	-2.63	DREB subfamily A-5 of ERF/AP2 transcription factor family
	*LFZN7F*	-1.42	HXXXD-type acyl-transferase family protein
	*Q9CK1R*	-2.30	Nicotinate phosphoribosyl transferase 1
	*IY6P5S*	-4.61	Protein kinase superfamily protein
	*GE1UVX*	-8.49	PI-PLC X domain-containing protein At5g67130-like
	*C2Y005*	1.71	Cyclin-dependent kinase inhibitor family protein
*qGPS.B02-1*	*G3JKAY*	1.39	Cyclic nucleotide gated channel 1
	*XD59WK*	1.53	Cyclic nucleotide gated channel 1
	*CF5AFL*	-1.52	Clustered mitochondria protein-like isoform X1
	*TG6F30*	-2.45	Myb transcription factor
	*MNZ0X1*	1.99	Dof zinc finger protein DOF3.6-like
	*YBIH8U*	2.12	Uncharacterized protein
	*H82QG0*	-2.16	Cytochrome P450 superfamily protein

*log_2_ FC* log_2_ Fold change value.

## 4 Discussion

Peanut produce aerial flowers but subterranean fruits (pods). Peanuts harvesting should put pods out of soil, which need strong gynophore-pod node and gynophore-branch node. Generally, the maximum break force of gynophore-branch node is much higher than that of gynophore-pod node. Thus, genetic improve gynophore-pod strength is meaningful to reduce peanut yield loss during mechanized harvesting.

Till now, genetic studies on gynophore-pod strength have not been reported. This may partly due to the challenges in obtaining accurate and consistent results for this trait. Gynophores dehydrate and become brittle just days after harvest. Considering that water content will alter gynophore-pod strength significantly, and dehydrated gynophore is brittle, which makes gynophore-pod strength assessment impossible. To address this, we tested various storage conditions to identify an optimal environment for preserving gynophore-pod samples, ensuring accurate measurements. Our findings revealed that unsealed storage led to rapid dehydration, affecting strength measurements. Sealed storage at 25 °C accelerated mildew, while -20 °C caused tissue damage, both severely impacting gynophore-pods strength measurements. However, the strength values for two parent samples (Xuzhou68-4 and Yuanza9102) did not differ significantly when stored sealed at 4 °C for 6 days. This demonstrates that accurate and consistent measurements can be achieved within 6 days of sample harvesting, which make large amount of gynophore-pod strength measurement possible. Additionally, the genetic effect of *qGPS.A05-1* and *qGPS.B02-1* could be detected repeatedly. It could be concluded that the storage condition used in this study was feasible in QTL analysis of gynophore-pod strength.

Despite a mere 1.28 N difference in parental trait means, this did not limit segregation variance and the power to detect QTLs in the RIL population. The similar results were also observed in shelling percentage trait using the same RIL population (Luo et al., 2017). Significant variances and transgressive segregations in present study indicated that the parents carry complementary alleles at several loci that were recombined in the progeny (Tanksley, 1993). For example, *qGPS.A05-1* had positive additive genetic effects, suggesting that the allele for increasing gynophore-pod strength came from the parent Xuzhou68-4. While the other QTLs we identified in this study had negative additive genetic effects, revealing that Yuanza9102 as the source of alleles that improve the gynophore-pod strength. This explained the transgressive segregation of gynophore-pod strength in the RIL population. Only *qGPS.A05-1* was identified with positive additive genetic effects, suggesting that additional QTLs for gynophore-pod strength remain unidentified. This may be attributed to the uneven distribution and limited number of SSR markers in the linkage map. For example, A04, A010, B03, B06, and B07 contain fewer than 10 markers each, with just 3 markers on A10 and B06. Additionally, only 8 linkage groups have over 50 markers (Luo et al., 2017).

The broad-sense heritability for gynophore-pod strength was estimated to be 0.77, indicating that genetic factors play a major role in the determination of this trait. But identification of QTLs for gynophore-pod strength was highly affected by environment. For example, 5 QTLs in this study were only detected in one environment. Variance analysis also indicated that environmental effects significantly influenced gynophore-pod strength. It is important to identified QTLs that consistently perform across different environments. In this study *qGPS.A05-1* was identified in all environments and *qGPS.B02-1* could be detected in two different environments. In addition, *qGPS.A05-1* and *qGPS.B02-1* were also detected using BLUE values. Although both QTLs have a relatively low PVE values, they remain valuable for improving gynophore-pod strength in peanut breeding.

To get insight into the molecular mechanisms underlying gynophore-pod strength in peanut, we achieved comparative transcriptome analysis of lines with extreme high and low gynophore-pod strength. GO and KEGG analyses showed significant enrichment of DEGs related to cell wall development. Furthermore, we found that several key genes in the phenylpropanoid biosynthesis pathway were notably downregulated in the low gynophore-pod strength line. Phenylpropanoid pathway provides precursors for lignin monolignol biosynthesis, which is crucial for lignin deposition in plant (Eudes et al., 2014; Ralph et al., 2019; Dong and Lin, 2021). Lignin content directly affects crop stem physical strength (Li et al., 2009; Peng et al., 2014). Abnormal lignin deposition in the abscission layer (Wu et al., 2017; Lv et al., 2018) or dehiscence zone (Dong et al., 2014) lead to shatter resistance. Additionally, anatomical analysis revealed that lignin content in gynophore-pod nodes was significantly higher in high gynophore-pod strength lines compared to low gynophore-pod strength lines. Altogether, these findings suggest that the high gynophore-pod strength observed in this study is largely due to enhanced lignin synthesis. Interestingly, no abscission layer, essential for seed shattering in cereal crops (Dong and Wang, 2015; Wu et al., 2017; Lv et al., 2018; Maity et al., 2021), was found at the gynophore-pod node in this study. Although lignin deposition significantly affects both gynophore-pod and seed-pedicel strength at their junctions, the molecular mechanisms governing gynophore-pod strength in peanuts may differ from those of seed-pedicel strength in cereal crops.

Combining transcriptome analysis and functional annotation information, we predicted candidate genes for the repeatedly detected QTLs *qGPS.A05-1* and *qGPS.B02-1*. *VJ8B3Q*, located within the *qGPS.A05-1* interval, is likely involved in regulating gynophore-pod strength. This gene encodes a DREB protein, from the ERF/AP2 transcription factor family. Many ERF/AP2 transcription factors involved in lignin development through the phenylpropanoid pathway. For example, NtERF13a promotes the phenylpropanoid compound contents and lignin in tobacco (Wang et al., 2023); overexpression *ScDREB10* and *OsERF71* increases lignin deposition by elevating the expression of phenylpropanoid-related genes (Lee et al., 2016; Liang et al., 2024). In the *qGPS.B02-1* interval, *H82QG0* which is a member of the CYP98A cytochrome P450 monooxygenase protein family was predicted as a candidate gene. Lignin biosynthesis requires three endoplasmic reticulum (ER)-resident cytochrome P450 monooxygenases, CYP73A5, CYP98A3 and CYP84A1, to establish the structural characteristics of its monomeric precursors (Boerjan et al., 2003). CYP98A3 is a crucial enzyme in the monolignol branch pathway, leading to the formation of G and/or S monolignols (Gou et al., 2018). Compared to the low gynophore-pod strength line, both *VJ8B3Q* and *H82QG0* were highly induced in the high gynophore-pod strength line. These results indicated that *VJ8B3Q* and *H82QG0* may be the key candidate genes for *qGPS.A05-1* and *qGPS.B02-1*, respectively. However, further investigations are needed to clarify the pathways underlying their regulation of gynophore-pod strength, as direct evidence of their involvement in cell wall component synthesis and deposition is still lacking.

## 5 Conclusion

This study examined the mechanism of gynophore-pod strength using QTL and transcriptome analysis. Two repeatedly detected QTLs, *qGPS.A05-1* and *qGPS.B02-1*, were mapped on chromosome A05 and B02, respectively. Transcriptome and anatomical analysis indicated that lignin biosynthesis was the main factor affecting gynophore-pod strength. Then we identified candidate genes for *qGPS.A05-1* and *qGPS.B02-1* by combining functional annotation with transcriptome data. These results will be useful for peanut breeding programs aiming to enhance peanut gynophore-pod strength for improved mechanized harvesting quality. This study established the foundation for the fine mapping and cloning the gene responsible for stable QTLs, offering gene resources for modifying peanut gynophore-pod traits.

## Data Availability

The datasets presented in this study can be found in online repositories. Transcriptome data (BioProject accession: PRJNA1182739) have been uploaded to the NCBI (https://www.ncbi.nlm.nih.gov/).
