# Supplementary material for: Integrating QTL mapping and transcriptome analysis to provide molecular insights into gynophore-pod strength in cultivated peanut (Arachis hypogaea L.)
Source: Front Plant Sci. 2024 Nov 19;15:1500281. doi: 10.3389/fpls.2024.1500281 (PMC11611583; doi:10.3389/fpls.2024.1500281)
Supplement: Supplementary file 1 [file DataSheet1.docx]

Supplementary Material

# Supplementary Figures and Tables

## Supplementary Figures


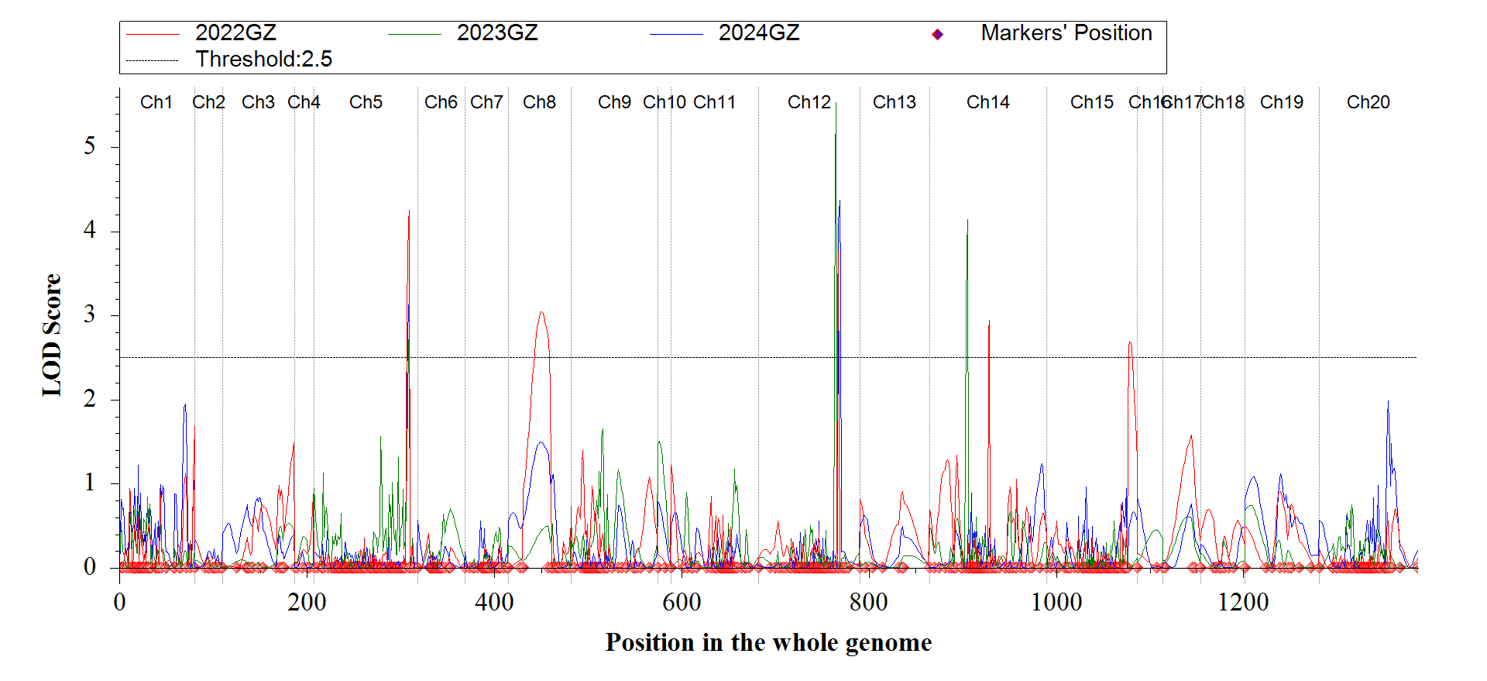


**Supplementary Figure 1.** Detection of gynophore-pod strength QTL in three different environments.


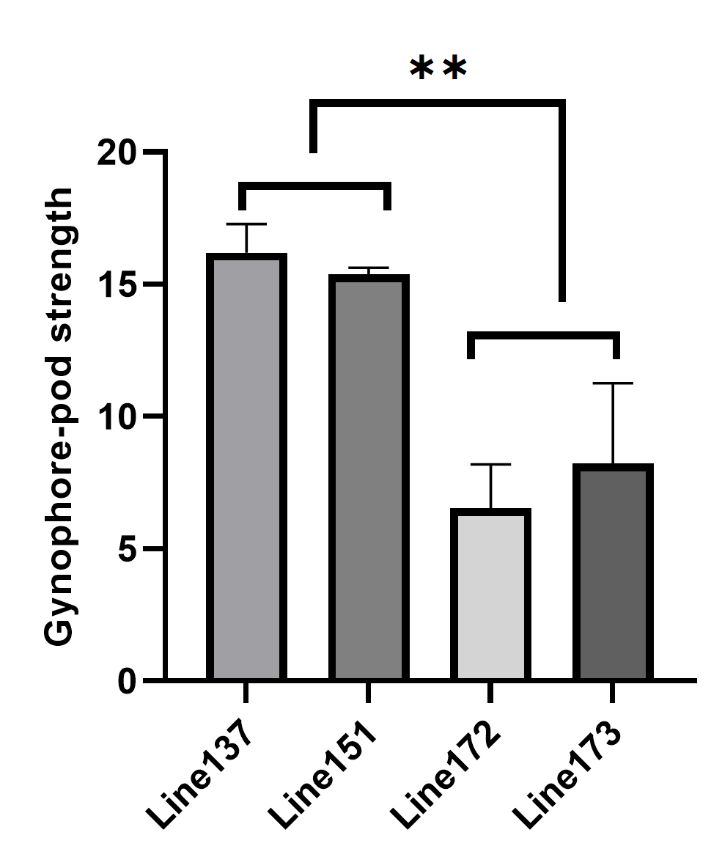


**Supplementary Figure 2.** Gynophore-pod strength exhibited significant difference between extreme lines.


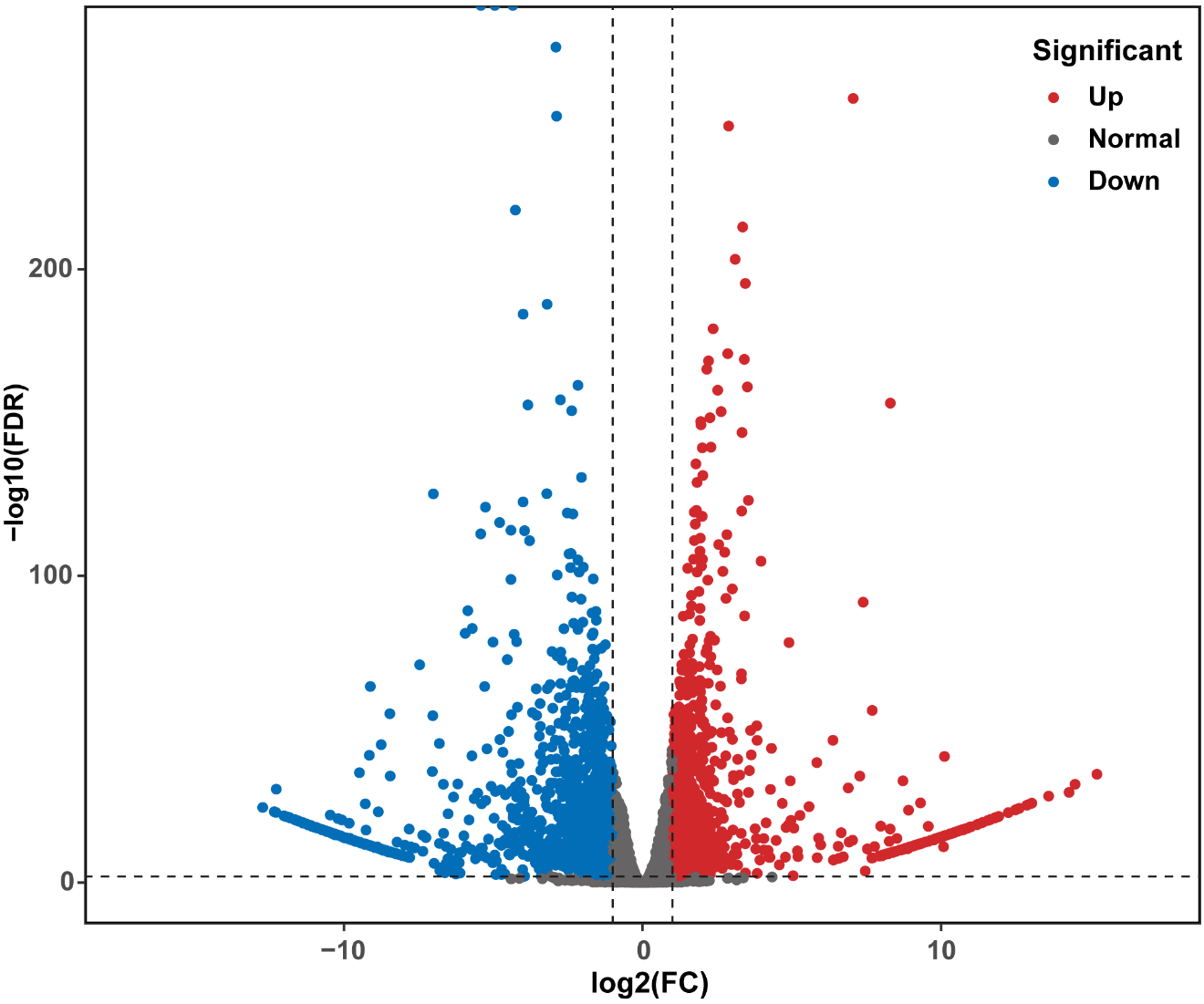


**Supplementary Figure 3.** Volcano plot of DEGs in line 137 and 173.


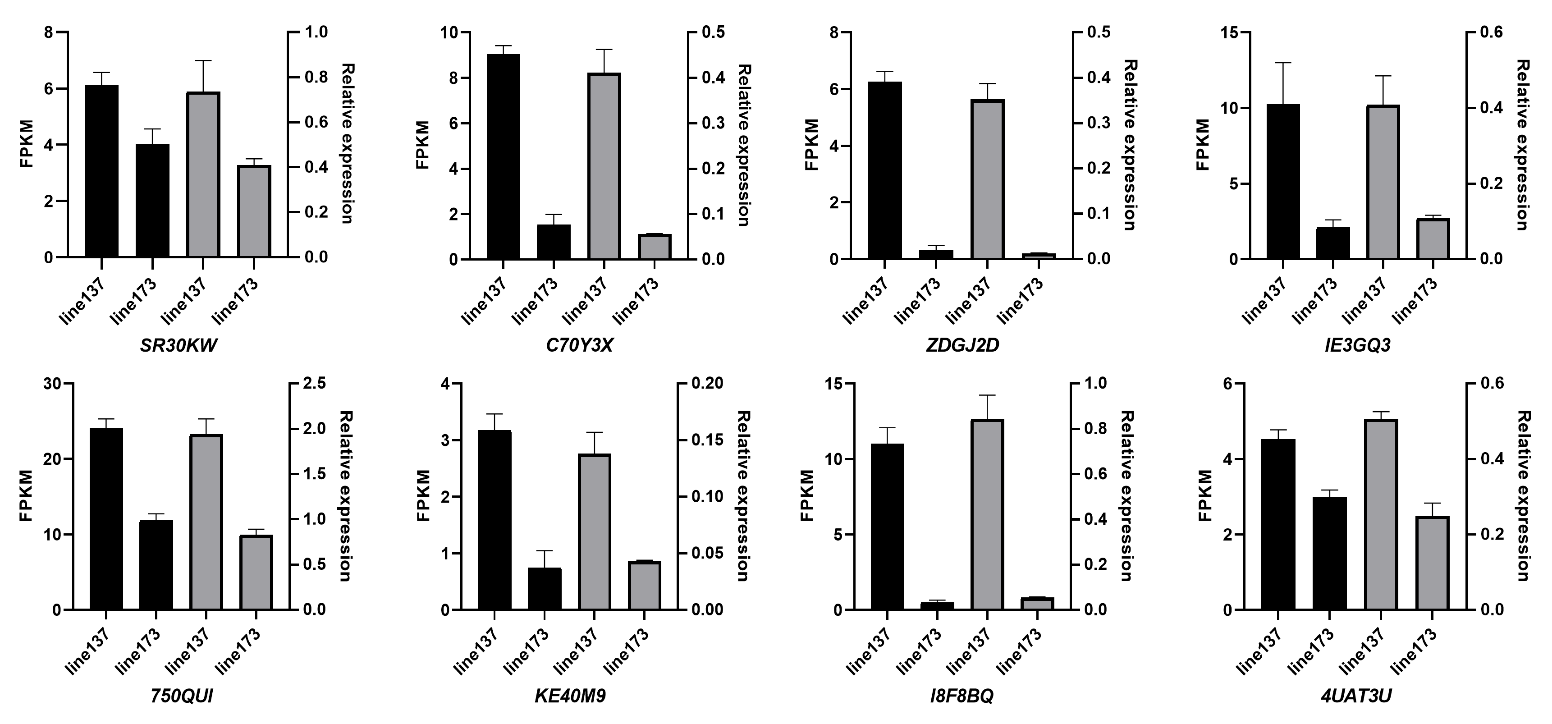


**Supplementary Figure 4.** Comparison of RT-PCR results with transcriptome results of extreme lines

## Supplementary Tables

**Supplementary Table 1** Primers used for RT-PCR

| Primer name | Sequence |
| --- | --- |
| qKE40M9-F | GGTTCTTGAGTCCTATGAAGGTTTT |
| qKE40M9-R | CTTA GCGGCGGTTG TATTCC |
| qI8F8BQ-F | ATGCGTAAGTACGTGTTCGTG |
| qI8F8BQ-R | TCACTCCTCG CCAGTCGCCA |
| qZDGJ2D-F | ACGATGAGGAGAAGCAGACCA |
| qZDGJ2D-R | GCAGG AGCAATATAG AAACCATCA |
| qIE3GQ3-F | GCAGTGAACTCTGCTGTGGC |
| qIE3GQ3-R | GCTCTCTACTTGAGACTTTATGGTG |
| qSR30KW-F | GAATTCAGTGAGAGGATTTGAGGT |
| qSR30KW-R | TCAA AGGGTGATGG CAAATTG |
| qC70Y3X-F | CTTCATTTTCATGATTGCTTTGTT |
| qC70Y3X-R | GCTCTCTACT TGGGACTTTA TGGTA |
| q750QUI-F | GCGTTTGTTCTTCCATGACTGT |
| q750QUI-R | GCGCAAGAAACAACGCCA |
| q4UAT3U-F | ATTCCATTATTTTCAATATGTCGTTAC |
| q4UAT3U-R | CTTGGAGTTCTACCACCAACAGC |

**Supplementary Table 2** The summary of the sequencing data

| Sample | Clean_Reads | Clean_Bases | Q20 (%) | Q30 (%) | GC (%) | Mapped Reads | Mapped raito (%) |
| --- | --- | --- | --- | --- | --- | --- | --- |
| L137-1 | 43517334 | 6.504E+09 | 99.22 | 97.31 | 44.39 | 41,454,567 | 95.26% |
| L137-2 | 42830666 | 6.386E+09 | 99.75 | 98.72 | 44.2 | 41,405,792 | 96.67% |
| L137-3 | 43173092 | 6.459E+09 | 99.09 | 97.16 | 44.59 | 41,033,395 | 95.04% |
| L173-1 | 46530474 | 6.932E+09 | 98.24 | 95.38 | 44.34 | 44,664,345 | 95.99% |
| L173-2 | 54421814 | 8.123E+09 | 98.66 | 96.26 | 44.35 | 52,259,891 | 96.03% |
| L173-3 | 47546858 | 7.088E+09 | 98.37 | 95.62 | 45.03 | 44,739,293 | 94.10% |

**Supplementary Table 3** Gene Ontology (GO) terms enrichment of DEGs in line 137 and line 173

| Category | GOID | Description | Gene  Ratio | Bg  Ratio | pvalue | padj | Count | Contained |
| --- | --- | --- | --- | --- | --- | --- | --- | --- |
| BP | GO:0009765 | photosynthesis, light harvesting | 0.0124 | 0.0015 | 2.486E-10 | 3.852E-07 | 14 | down |
| BP | GO:0071555 | cell wall organization | 0.0381 | 0.0159 | 1.132E-07 | 8.505E-05 | 43 | up&down |
| BP | GO:0015979 | photosynthesis | 0.0248 | 0.0082 | 1.647E-07 | 8.505E-05 | 28 | down |
| BP | GO:0018298 | protein-chromophore linkage | 0.0151 | 0.0035 | 3.347E-07 | 0.0001297 | 17 | up&down |
| BP | GO:0042546 | cell wall biogenesis | 0.0186 | 0.0055 | 7.643E-07 | 0.000221 | 21 | up&down |
| BP | GO:0005975 | carbohydrate metabolic process | 0.0736 | 0.0426 | 8.557E-07 | 0.000221 | 83 | up&down |
| BP | GO:0009058 | biosynthetic process | 0.0629 | 0.035 | 1.257E-06 | 0.0002782 | 71 | up&down |
| BP | GO:0007165 | signal transduction | 0.0691 | 0.0413 | 5.586E-06 | 0.0009949 | 78 | up&down |
| BP | GO:0042549 | photosystem II stabilization | 0.0035 | 0.0002 | 5.832E-06 | 0.0009949 | 4 | down |
| BP | GO:0006952 | defense response | 0.0505 | 0.0274 | 6.421E-06 | 0.0009949 | 57 | up&down |
| BP | GO:0030244 | cellulose biosynthetic process | 0.0151 | 0.0048 | 2.622E-05 | 0.003533 | 17 | up&down |
| BP | GO:0010411 | xyloglucan metabolic process | 0.0115 | 0.003 | 2.736E-05 | 0.003533 | 13 | up&down |
| BP | GO:0006270 | DNA replication initiation | 0.008 | 0.0016 | 5.842E-05 | 0.0069627 | 9 | up |
| BP | GO:0051865 | protein autoubiquitination | 0.008 | 0.0017 | 7.323E-05 | 0.0081044 | 9 | up&down |
| BP | GO:0042744 | hydrogen peroxide catabolic process | 0.0186 | 0.0076 | 0.0001361 | 0.0140552 | 21 | up&down |
| BP | GO:2000022 | regulation of jasmonic acid mediated signaling pathway | 0.0071 | 0.0014 | 0.0001547 | 0.0149768 | 8 | up |
| BP | GO:0010207 | photosystem II assembly | 0.0044 | 0.0006 | 0.0002641 | 0.0240675 | 5 | down |
| BP | GO:0006979 | response to oxidative stress | 0.0319 | 0.0172 | 0.0002974 | 0.0255988 | 36 | up&down |
| BP | GO:0009635 | response to herbicide | 0.0044 | 0.0006 | 0.0003941 | 0.0321431 | 5 | down |
| BP | GO:0042537 | benzene-containing compound metabolic process | 0.0053 | 0.001 | 0.0005291 | 0.0409895 | 6 | down |
| BP | GO:0009834 | plant-type secondary cell wall biogenesis | 0.0044 | 0.0007 | 0.0005673 | 0.0418612 | 5 | down |
| BP | GO:0009738 | abscisic acid-activated signaling pathway | 0.0124 | 0.0046 | 0.0006303 | 0.0443892 | 14 | down |
| BP | GO:0018973 | trinitrotoluene metabolic process | 0.0035 | 0.0004 | 0.0009642 | 0.047628 | 4 | down |
| BP | GO:0018974 | 2,4,6-trinitrotoluene metabolic process | 0.0035 | 0.0004 | 0.0009642 | 0.047628 | 4 | down |
| BP | GO:0019326 | nitrotoluene metabolic process | 0.0035 | 0.0004 | 0.0009642 | 0.047628 | 4 | down |
| BP | GO:0046256 | 2,4,6-trinitrotoluene catabolic process | 0.0035 | 0.0004 | 0.0009642 | 0.047628 | 4 | down |
| BP | GO:0046260 | trinitrotoluene catabolic process | 0.0035 | 0.0004 | 0.0009642 | 0.047628 | 4 | down |
| BP | GO:0046263 | nitrotoluene catabolic process | 0.0035 | 0.0004 | 0.0009642 | 0.047628 | 4 | down |
| BP | GO:0072490 | toluene-containing compound metabolic process | 0.0035 | 0.0004 | 0.0009642 | 0.047628 | 4 | down |
| BP | GO:0072491 | toluene-containing compound catabolic process | 0.0035 | 0.0004 | 0.0009642 | 0.047628 | 4 | down |
| BP | GO:0071215 | cellular response to abscisic acid stimulus | 0.0071 | 0.0019 | 0.0010442 | 0.047628 | 8 | down |
| BP | GO:0097306 | cellular response to alcohol | 0.0071 | 0.0019 | 0.0010442 | 0.047628 | 8 | down |
| BP | GO:0051259 | protein complex oligomerization | 0.0098 | 0.0033 | 0.001059 | 0.047628 | 11 | up&down |
| BP | GO:0046928 | regulation of neurotransmitter secretion | 0.0027 | 0.0002 | 0.0011025 | 0.047628 | 3 | up |
| BP | GO:0051588 | regulation of neurotransmitter transport | 0.0027 | 0.0002 | 0.0011025 | 0.047628 | 3 | up |
| BP | GO:0016998 | cell wall macromolecule catabolic process | 0.0053 | 0.0011 | 0.0011066 | 0.047628 | 6 | up&down |
| BP | GO:0071396 | cellular response to lipid | 0.0133 | 0.0056 | 0.0015838 | 0.0642595 | 15 | up&down |
| BP | GO:0010923 | negative regulation of phosphatase activity | 0.0053 | 0.0012 | 0.0017003 | 0.0642595 | 6 | down |
| BP | GO:0032515 | negative regulation of phosphoprotein phosphatase activity | 0.0053 | 0.0012 | 0.0017003 | 0.0642595 | 6 | down |
| BP | GO:0035305 | negative regulation of dephosphorylation | 0.0053 | 0.0012 | 0.0017003 | 0.0642595 | 6 | down |
| BP | GO:0035308 | negative regulation of protein dephosphorylation | 0.0053 | 0.0012 | 0.0017003 | 0.0642595 | 6 | down |
| BP | GO:0071229 | cellular response to acid chemical | 0.0151 | 0.0068 | 0.0017882 | 0.0659703 | 17 | up&down |
| BP | GO:1902882 | regulation of response to oxidative stress | 0.0044 | 0.0008 | 0.0018639 | 0.0671546 | 5 | up&down |
| BP | GO:1901701 | cellular response to oxygen-containing compound | 0.0204 | 0.0105 | 0.001907 | 0.0671546 | 23 | up&down |
| BP | GO:0006032 | chitin catabolic process | 0.0053 | 0.0012 | 0.0020751 | 0.0707339 | 6 | up&down |
| BP | GO:0015976 | carbon utilization | 0.0035 | 0.0005 | 0.0020999 | 0.0707339 | 4 | down |
| BP | GO:0015995 | chlorophyll biosynthetic process | 0.0062 | 0.0017 | 0.0022648 | 0.0746643 | 7 | down |
| BP | GO:1905775 | negative regulation of DNA helicase activity | 0.0018 | 0.0001 | 0.002419 | 0.0780866 | 2 | up |
| BP | GO:0080134 | regulation of response to stress | 0.0168 | 0.0083 | 0.0026989 | 0.0853443 | 19 | up&down |
| BP | GO:0045492 | xylan biosynthetic process | 0.0044 | 0.0009 | 0.0030056 | 0.0931414 | 5 | down |
| BP | GO:0001505 | regulation of neurotransmitter levels | 0.0027 | 0.0003 | 0.0035815 | 0.1047073 | 3 | up |
| BP | GO:0060776 | simple leaf morphogenesis | 0.0027 | 0.0003 | 0.0035815 | 0.1047073 | 3 | up |
| BP | GO:0080037 | negative regulation of cytokinin-activated signaling pathway | 0.0027 | 0.0003 | 0.0035815 | 0.1047073 | 3 | down |
| BP | GO:0031400 | negative regulation of protein modification process | 0.0071 | 0.0023 | 0.0036695 | 0.1052919 | 8 | up&down |
| BP | GO:0009611 | response to wounding | 0.0124 | 0.0055 | 0.0039307 | 0.1107373 | 14 | up&down |
| BP | GO:0009112 | nucleobase metabolic process | 0.0044 | 0.001 | 0.0045806 | 0.1267403 | 5 | up&down |
| BP | GO:0043455 | regulation of secondary metabolic process | 0.0035 | 0.0007 | 0.0051453 | 0.1344927 | 4 | down |
| BP | GO:0010563 | negative regulation of phosphorus metabolic process | 0.0062 | 0.0019 | 0.0053261 | 0.1344927 | 7 | up&down |
| BP | GO:0045936 | negative regulation of phosphate metabolic process | 0.0062 | 0.0019 | 0.0053261 | 0.1344927 | 7 | up&down |
| BP | GO:0035435 | phosphate ion transmembrane transport | 0.0027 | 0.0003 | 0.0055217 | 0.1344927 | 3 | down |
| BP | GO:0050804 | modulation of chemical synaptic transmission | 0.0027 | 0.0003 | 0.0055217 | 0.1344927 | 3 | up |
| BP | GO:0099177 | regulation of trans-synaptic signaling | 0.0027 | 0.0003 | 0.0055217 | 0.1344927 | 3 | up |
| BP | GO:0042631 | cellular response to water deprivation | 0.0044 | 0.001 | 0.0055551 | 0.1344927 | 5 | up&down |
| BP | GO:0071462 | cellular response to water stimulus | 0.0044 | 0.001 | 0.0055551 | 0.1344927 | 5 | up&down |
| BP | GO:0009768 | photosynthesis, light harvesting in photosystem I | 0.0018 | 0.0001 | 0.0070193 | 0.1673267 | 2 | down |
| BP | GO:0048583 | regulation of response to stimulus | 0.0346 | 0.023 | 0.0078259 | 0.1792339 | 39 | up&down |
| BP | GO:2000030 | regulation of response to red or far red light | 0.0044 | 0.0011 | 0.0079263 | 0.1792339 | 5 | down |
| BP | GO:0019747 | regulation of isoprenoid metabolic process | 0.0027 | 0.0004 | 0.0079815 | 0.1792339 | 3 | down |
| BP | GO:0080036 | regulation of cytokinin-activated signaling pathway | 0.0027 | 0.0004 | 0.0079815 | 0.1792339 | 3 | down |
| BP | GO:0001560 | regulation of cell growth by extracellular stimulus | 0.0035 | 0.0007 | 0.0082948 | 0.1810227 | 4 | up&down |
| BP | GO:0019428 | allantoin biosynthetic process | 0.0035 | 0.0007 | 0.0082948 | 0.1810227 | 4 | up&down |
| BP | GO:0051289 | protein homotetramerization | 0.0044 | 0.0012 | 0.0093413 | 0.2010295 | 5 | up&down |
| CC | GO:0009522 | photosystem I | 1.41% | 0.24% | 3.54E-10 | 9.83E-08 | 19 | down |
| CC | GO:0009523 | photosystem II | 1.19% | 0.22% | 2.41E-08 | 3.34E-06 | 16 | down |
| CC | GO:0009538 | photosystem I reaction center | 0.59% | 0.05% | 1.30E-07 | 1.20E-05 | 8 | down |
| CC | GO:0048046 | apoplast | 2.59% | 0.98% | 1.80E-07 | 1.25E-05 | 35 | up&down |
| CC | GO:0042555 | MCM complex | 0.67% | 0.08% | 5.77E-07 | 3.21E-05 | 9 | up |
| CC | GO:0005618 | cell wall | 2.81% | 1.21% | 1.34E-06 | 6.22E-05 | 38 | up&down |
| CC | GO:0009654 | photosystem II oxygen evolving complex | 0.81% | 0.21% | 9.88E-05 | 0.003924 | 11 | down |
| CC | GO:0009535 | chloroplast thylakoid membrane | 1.78% | 0.91% | 0.0014768 | 0.0513002 | 24 | down |
| CC | GO:0031225 | anchored component of membrane | 0.74% | 0.27% | 0.003012 | 0.0930016 | 10 | up&down |
| CC | GO:0009505 | plant-type cell wall | 0.67% | 0.26% | 0.0073214 | 0.2033424 | 9 | up&down |
| MF | GO:0043531 | ADP binding | 5.51% | 2.72% | 6.94E-13 | 2.14E-10 | 115 | up&down |
| MF | GO:0016747 | transferase activity, transferring acyl groups other than amino-acyl groups | 2.87% | 1.03% | 7.45E-13 | 2.14E-10 | 60 | up&down |
| MF | GO:0020037 | heme binding | 4.93% | 2.36% | 1.67E-12 | 3.20E-10 | 103 | up&down |
| MF | GO:0004497 | monooxygenase activity | 3.26% | 1.30% | 4.15E-12 | 5.97E-10 | 68 | up&down |
| MF | GO:0016705 | oxidoreductase activity, acting on paired donors, with incorporation or reduction of molecular oxygen | 3.69% | 1.72% | 3.17E-10 | 3.65E-08 | 77 | up&down |
| MF | GO:0004553 | hydrolase activity, hydrolyzing O-glycosyl compounds | 3.02% | 1.33% | 1.48E-09 | 1.42E-07 | 63 | up&down |
| MF | GO:0005506 | iron ion binding | 3.93% | 2.05% | 1.70E-08 | 1.40E-06 | 82 | up&down |
| MF | GO:0008194 | UDP-glycosyltransferase activity | 2.01% | 0.83% | 1.18E-07 | 8.51E-06 | 42 | up&down |
| MF | GO:0016760 | cellulose synthase (UDP-forming) activity | 0.77% | 0.20% | 3.34E-06 | 0.000214 | 16 | up&down |
| MF | GO:0016168 | chlorophyll binding | 0.77% | 0.20% | 3.96E-06 | 0.0002278 | 16 | down |
| MF | GO:0010242 | oxygen evolving activity | 0.19% | 0.01% | 7.10E-06 | 0.0003718 | 4 | down |
| MF | GO:0016762 | xyloglucan:xyloglucosyl transferase activity | 0.62% | 0.15% | 9.49E-06 | 0.0004555 | 13 | up&down |
| MF | GO:0016491 | oxidoreductase activity | 4.69% | 3.08% | 2.39E-05 | 0.0010586 | 98 | up&down |
| MF | GO:0031406 | carboxylic acid binding | 0.48% | 0.11% | 8.02E-05 | 0.0030769 | 10 | up&down |
| MF | GO:0043177 | organic acid binding | 0.48% | 0.11% | 8.02E-05 | 0.0030769 | 10 | up&down |
| MF | GO:0015369 | calcium:proton antiporter activity | 0.24% | 0.03% | 0.0001301 | 0.0046829 | 5 | up&down |
| MF | GO:0019840 | isoprenoid binding | 0.29% | 0.05% | 0.0003904 | 0.0132218 | 6 | down |
| MF | GO:0030246 | carbohydrate binding | 1.53% | 0.81% | 0.00044 | 0.0140734 | 32 | up&down |
| MF | GO:0015267 | channel activity | 0.67% | 0.24% | 0.0005094 | 0.0154362 | 14 | down |
| MF | GO:0016597 | amino acid binding | 0.29% | 0.05% | 0.0006875 | 0.0197921 | 6 | up |
| MF | GO:0030145 | manganese ion binding | 0.48% | 0.15% | 0.000818 | 0.022428 | 10 | down |
| MF | GO:0010427 | abscisic acid binding | 0.57% | 0.20% | 0.0009006 | 0.0235709 | 12 | down |
| MF | GO:0004089 | carbonate dehydratase activity | 0.29% | 0.06% | 0.0017765 | 0.0427373 | 6 | down |
| MF | GO:0005199 | structural constituent of cell wall | 0.24% | 0.04% | 0.0017814 | 0.0427373 | 5 | up |
| MF | GO:0019212 | phosphatase inhibitor activity | 0.29% | 0.07% | 0.0021853 | 0.050331 | 6 | down |
| MF | GO:0004601 | peroxidase activity | 1.25% | 0.68% | 0.0023256 | 0.0515016 | 26 | up&down |
| MF | GO:0043178 | alcohol binding | 0.29% | 0.07% | 0.0026611 | 0.0545331 | 6 | down |
| MF | GO:0003678 | DNA helicase activity | 0.53% | 0.20% | 0.0026723 | 0.0545331 | 11 | up&down |
| MF | GO:0004864 | protein phosphatase inhibitor activity | 0.57% | 0.23% | 0.0027466 | 0.0545331 | 12 | down |
| MF | GO:0008171 | O-methyltransferase activity | 0.72% | 0.32% | 0.0029831 | 0.0572555 | 15 | up&down |
| MF | GO:0033293 | monocarboxylic acid binding | 0.29% | 0.07% | 0.0032105 | 0.059632 | 6 | down |
| MF | GO:0042562 | hormone binding | 0.29% | 0.07% | 0.0038402 | 0.069098 | 6 | down |
| MF | GO:0016830 | carbon-carbon lyase activity | 0.29% | 0.08% | 0.0045568 | 0.0787723 | 6 | up&down |
| MF | GO:0045735 | nutrient reservoir activity | 0.48% | 0.18% | 0.0046515 | 0.0787723 | 10 | down |
| MF | GO:0015020 | glucuronosyltransferase activity | 0.14% | 0.02% | 0.006336 | 0.1042342 | 3 | down |
| MF | GO:0004649 | poly(ADP-ribose) glycohydrolase activity | 0.10% | 0.01% | 0.0077269 | 0.1235858 | 2 | up |
| MF | GO:0038023 | signaling receptor activity | 0.72% | 0.36% | 0.0082126 | 0.1253199 | 15 | up&down |
| MF | GO:0004568 | chitinase activity | 0.29% | 0.09% | 0.0084287 | 0.1253199 | 6 | up&down |
| MF | GO:0003714 | transcription corepressor activity | 0.38% | 0.14% | 0.0086541 | 0.1253199 | 8 | up |
| MF | GO:0005375 | copper ion transmembrane transporter activity | 0.14% | 0.02% | 0.0091413 | 0.1253199 | 3 | up |
| MF | GO:0008107 | galactoside 2-alpha-L-fucosyltransferase activity | 0.14% | 0.02% | 0.0091413 | 0.1253199 | 3 | down |
| MF | GO:0047216 | inositol 3-alpha-galactosyltransferase activity | 0.14% | 0.02% | 0.0091413 | 0.1253199 | 3 | down |
| MF | GO:0016812 | hydrolase activity, acting on carbon-nitrogen (but not peptide) bonds, in cyclic amides | 0.19% | 0.04% | 0.0098356 | 0.12585 | 4 | up&down |
| MF | GO:0033971 | hydroxyisourate hydrolase activity | 0.19% | 0.04% | 0.0098356 | 0.12585 | 4 | up&down |
| MF | GO:0051997 | 2-oxo-4-hydroxy-4-carboxy-5-ureidoimidazoline decarboxylase activity | 0.19% | 0.04% | 0.0098356 | 0.12585 | 4 | up&down |

**Supplementary Table 4** KEGG pathway enrichment of DEGs in line 137 and line 173

| ID | Pathway | GeneRatio | BgRatio | enrich_factor | pvalue | qvalue | gene_number | Contained |
| --- | --- | --- | --- | --- | --- | --- | --- | --- |
| ko04712 | Circadian rhythm - plant | 5.58% | 1.32% | 4.22 | 7.13E-25 | 7.81E-23 | 69 | up&down |
| ko00941 | Flavonoid biosynthesis | 4.20% | 1.02% | 4.13 | 1.34E-18 | 7.31E-17 | 52 | up&down |
| ko00196 | Photosynthesis - antenna proteins | 1.37% | 0.15% | 9.33 | 2.51E-13 | 9.16E-12 | 17 | up&down |
| ko00940 | Phenylpropanoid biosynthesis | 5.82% | 3.10% | 1.88 | 2.03E-07 | 5.55E-06 | 72 | up&down |
| ko00908 | Zeatin biosynthesis | 1.62% | 0.51% | 3.15 | 4.81E-06 | 0.0001054 | 20 | up&down |
| ko00902 | Monoterpenoid biosynthesis | 1.13% | 0.29% | 3.9 | 1.04E-05 | 0.0001899 | 14 | up&down |
| ko04075 | Plant hormone signal transduction | 9.22% | 6.73% | 1.37 | 0.0003749 | 0.0058626 | 114 | up&down |
| ko00592 | alpha-Linolenic acid metabolism | 1.46% | 0.68% | 2.15 | 0.0018488 | 0.0252988 | 18 | up&down |
| ko00195 | Photosynthesis | 2.02% | 1.12% | 1.81 | 0.0032348 | 0.0393474 | 25 | down |
| ko04626 | Plant-pathogen interaction | 10.19% | 8.09% | 1.26 | 0.0040503 | 0.0437167 | 126 | up&down |
| ko00460 | Cyanoamino acid metabolism | 1.94% | 1.09% | 1.79 | 0.0043927 | 0.0437167 | 24 | up&down |
| ko00904 | Diterpenoid biosynthesis | 1.13% | 0.53% | 2.15 | 0.0055543 | 0.0506711 | 14 | up&down |
| ko00909 | Sesquiterpenoid and triterpenoid biosynthesis | 1.13% | 0.56% | 2.02 | 0.0094822 | 0.07985 | 14 | up&down |
| ko04016 | MAPK signaling pathway - plant | 5.90% | 4.49% | 1.31 | 0.0102856 | 0.0804286 | 73 | up&down |
| ko00500 | Starch and sucrose metabolism | 6.06% | 4.69% | 1.29 | 0.0133628 | 0.0975249 | 75 | up&down |
| ko00250 | Alanine, aspartate and glutamate metabolism | 1.21% | 0.66% | 1.84 | 0.0170961 | 0.1169733 | 15 | up&down |
| ko00402 | Benzoxazinoid biosynthesis | 0.57% | 0.24% | 2.32 | 0.0302205 | 0.1946086 | 7 | up&down |
| ko00943 | Isoflavonoid biosynthesis | 0.97% | 0.54% | 1.79 | 0.0367046 | 0.2232329 | 12 | up&down |
| ko00430 | Taurine and hypotaurine metabolism | 0.24% | 0.06% | 3.84 | 0.0399984 | 0.2304618 | 3 | down |
| ko00591 | Linoleic acid metabolism | 0.57% | 0.26% | 2.14 | 0.0446103 | 0.2441825 | 7 | up&down |
| ko00052 | Galactose metabolism | 2.26% | 1.62% | 1.4 | 0.0472049 | 0.2460808 | 28 | up&down |
